# Hydropneumothorax in Children: A Rare Complication of a Bacterial Pneumonia

**DOI:** 10.1155/2016/8097105

**Published:** 2016-05-09

**Authors:** Carolina Ortega, Carla Gonzales, Manuel E. Soto-Martinez, Adriana Yock-Corrales

**Affiliations:** ^1^Emergency Department, Hospital Nacional de Niños “Dr. Carlos Sáenz Herrera”, 1654-1000 San José, Costa Rica; ^2^Respiratory Department, Hospital Nacional de Niños “Dr. Carlos Sáenz Herrera”, 1654-1000 San José, Costa Rica

## Abstract

Hydropneumothorax is an uncommon presentation of a complicated pneumonia, and very few cases in the pediatric population have been reported. This is a case of a 5-month-old patient who presented to the emergency department (ED) with a three-day history of fever, diarrhea, and respiratory distress. His initial assessment suggested a lower respiratory tract infection and because of his respiratory distress and hypoxia a chest X-ray was performed. Other clinical information and radiologic studies will be discussed further, but his chest X-ray suggested a right-sided hydropneumothorax secondary to a complicated pneumonia. He completed 12 days of IV antibiotic treatment and required a chest tube for drainage. Patient was discharged home with a full recovery.

## 1. Introduction

A hydropneumothorax is a rare radiologic finding which consists in the concurrent presence of both free fluid and air within the pleural space [1]. It can occur secondary to various situations such as thoracocentesis, thoracic trauma, esophagopleural fistula, or bronchopleural fistula. It is an uncommon presentation in the pediatric population and has been described more frequently secondary to a complicated pneumonia with bronchopleural fistula. Worldwide, this is a rare condition in children and just a few case reports have been published [2, 3].

Radiological investigations are the key to confirm the diagnosis and a high clinical suspicion is needed. As it was mentioned, the etiologies are diverse and can be associated with a complication of an invasive procedure, secondary to malignancy, infection, or rheumatologic disease [4–6]. The treatment will be determined according to the etiology found. The main aim of this case is to illustrate a rare radiologic finding of a relatively common condition in children.

## 2. Case Presentation

A 5-month-old patient presented to the emergency department at the National Children's Hospital with a three-day history of fever, diarrhea, and respiratory distress. He had a past medical history of prematurity of 28 weeks of gestational age from a twin pregnancy. He received surfactant at birth and did not require mechanical ventilation afterward. He was discharged home at day 30th after birth with no oxygen dependency. He was admitted for 1 month after being discharged from hospital for weight gain and problems with feeding intolerance. The patient was discharged again home in a good condition.

He had a previous consultation to a rural hospital 3 days prior to the ED presentation where the physician diagnosed pharyngitis and prescribed antibiotic treatment with amoxicillin for 7 days. The patient did not improve and he presented to the ED because of persistent fever.

On physical examination the patient was irritable, crying without signs of dehydration and without signs of hemodynamic instability. He had mild nasal flaring and intercostal recession with decreased air entry in the right lung field. No crackles or wheeze was found. He had normal heart sounds, and abdominal exploration was normal.

Laboratory investigations reported a CBC with 18,780 leukocytes/mm^3^ (63% neutrophils, 22% lymphocytes), hemoglobin 9.46 gr/dL, and platelets 565,000/mm^3^. Blood gases reported a pH of 7.34, pCO_2_ of 42 mmHg, pO_2_2 of 38.2 mmHg, EB of −3.58, HCO_3_^−^ of 22 mEq/L, BUN of 4 mg/dL, creatinine of 0.26 mg/dL, and a CRP of 224 IU/L. Two blood cultures drawn at admission were negative; and stool sample was normal with negative latex for rotavirus and adenovirus.

**Figure 1:**
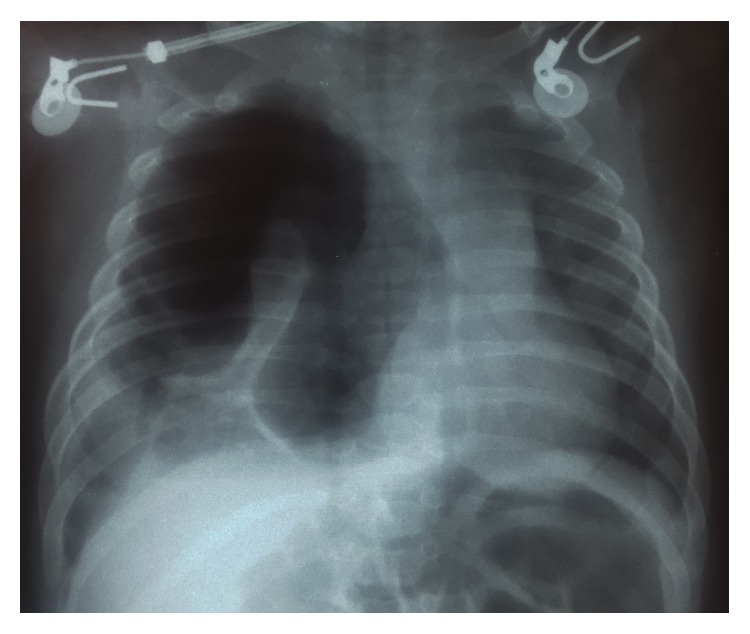
Chest X-ray on admission.

A chest X-ray was performed after finding the patient with respiratory distress. Chest X-ray showed a radiolucent image, loculated right lung, with a partial collapse of the lung showing images suggesting pleural adhesions, probably in relation to a loculated pneumothorax. Transmediastinal herniation of pneumothorax was seen. The diaphragm was visible without any intestine seen inside the chest. A pneumothorax was diagnosed in association with air-fluid level consistent with hydropneumothorax (Figure 1).

**Figure 2:**
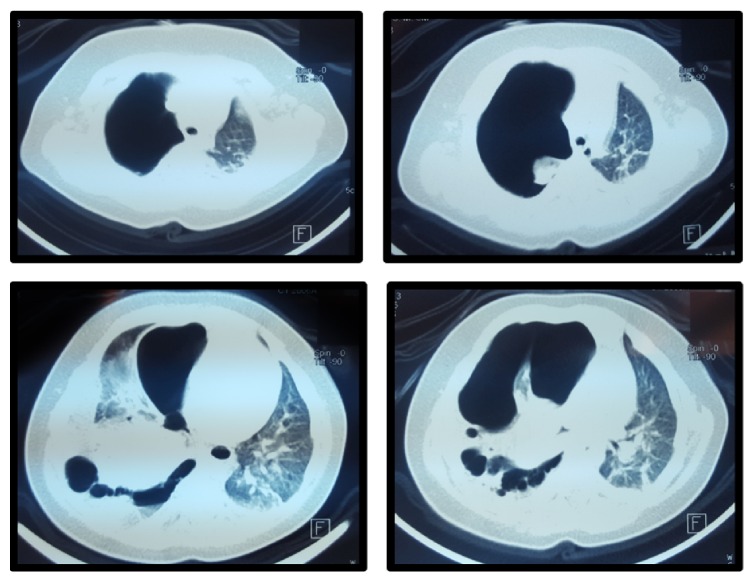
Chest CT scan.

CT of the chest was performed showing an extensive loculated right hydropneumothorax, with almost complete collapse of the left lung. The pattern that presented the collapsed right lung was atypical, with pleural adhesions to the anterior chest wall; and small amount of pleural fluid distributed to the anterolateral and posterior side of the hemithorax. Within the collapsed lung at least 3 bullae were observed (Figure 2).

**Figure 3:**
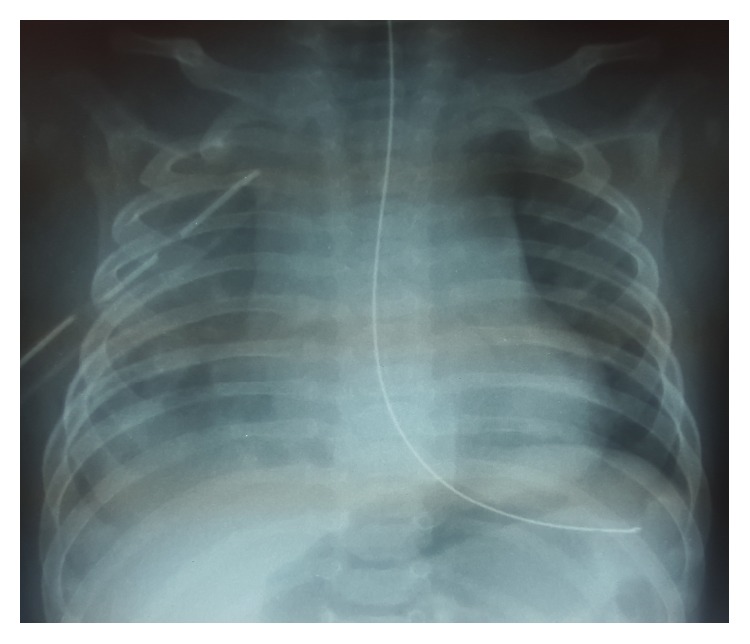
Chest X-ray after chest drainage.

**Figure 4:**
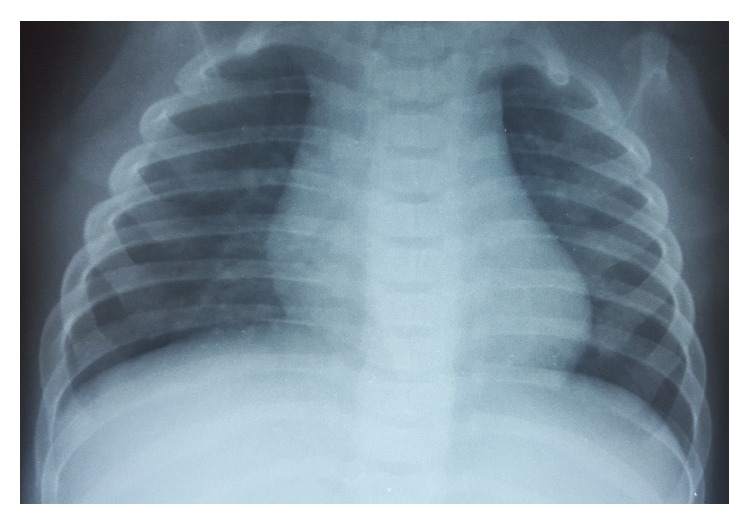
Normal chest X-ray on follow-up.

Hydropneumothorax was treated with a chest tube. Chest X-ray after the procedure showed a complete resolution of the hydropneumothorax (Figure 3). The culture of the pleural effusion reported a methicillin-resistant* Staphylococcus aureus* (MRSA). Oxygen therapy with nasal prongs was required for 12 days and intravenous antibiotic regimen with Clindamycin for 14 days was administered. The patient was discharged with antibiotic treatment with trimethoprim for seven more days. On follow-up, one month later, the patient was asymptomatic with a normal chest X-ray (Figure 4).

## 3. Discussion

Hydropneumothorax is a rare variant type of a pneumothorax. It consists of both free fluid and air within the pleural space. In the pediatric population, hydropneumothorax has been associated with rupture of a diaphragmatic hernia, after thoracocentesis, and trauma and with infections such as tuberculosis [1, 7, 8].

Hydropneumothorax may be a complication of an invasive procedure such as a transbronchial biopsy, chest tube placement, or thoracocentesis. Other etiologies include malignancy, after a chest trauma, secondary to a pneumonectomy, infection, pulmonary infarction, cystic lung disease, obstructive lung disease, and rarely connective tissue disorders such as dermatomyositis [5, 9–11]. In the literature we did not found any case report associated with complicated pneumonia caused by MRSA.

Hydropneumothorax and pleural effusions are typically presented with sudden onset of unilateral thoracic pain and dyspnea. Asymmetrical expansion of the hemithorax is usually observed associated with decreased air entry. The diagnosis of hydropneumothorax is suspected by chest X-ray and confirmed by computed tomography. In the chest X-ray, the pleural effusion has a characteristic feature and is a meniscus along the chest wall with obliteration of the hemidiaphragm. In a hydropneumothorax, a meniscus is not observed because the trapped air leads to an increase in intrathoracic pressure that obliterates the fluid interface. An air-fluid level on the chest X-ray may be an important clue to suspect that a pneumothorax exists [1].

The diagnosis of a diaphragmatic rupture requires a high index of clinical suspicion and careful look of the chest radiograph. Radiological features that suggest the possibility of a diaphragmatic rupture include elevated hemidiaphragm, irregular diaphragmatic outline, gas bubble in the chest, nasogastric tube in the chest, and compression atelectasis of the lower lobe [7].

Ultrasound has been used in the past to visualize the characteristics of a hydropneumothorax. The ultrasonographic signs seen in one study were gassy effusion above the pleural fluid, the disappearance of the “gliding sign” described as back-and-forth respiratory movements, and the “curtain sign” that is the movement of air fluid level. Also a “polymicrobullous” image caused by air micro bubbles within the fluid effusion might be observed. Although a good descriptive method, it is not a good one to determine the nature of a hydropneumothorax [12, 13].

There are different protocols in the management of simple pleural effusion and hydropneumothorax according to the severity. Initial treatment should focus on the management of airway, breathing, and circulation. Patients with significant respiratory distress require in many cases a definite airway and an aggressive management of the underlying disease. Oxygen may increase the rate of hydropneumothorax reabsorption, with a fourfold effect demonstrated in the presence of a pneumothorax greater than 30% of the lung field [14].

The difference between hydropneumothorax and a simple pleural effusion is necessary because appropriate treatment of a hydropneumothorax often requires specific site placement of two chest tubes, one to drain the fluid and the other to remove the air. On the other hand, simple pleural effusion often requires a single chest tube [1].

This case is an uncommon presentation of hydropneumothorax. Few cases in the pediatric population have been reported. Hydropneumothorax is present most typically in newborns and adolescents. The diagnosis is suspected by X-ray and computed tomography is the method of choice. Ultrasound is not the best method to determine the nature of a hydropneumothorax. Initial treatment in the pediatric population should include assessment of the airway, cardiac monitoring, and immediate stabilization. Chest tube insertion is necessary to drain the fluid and remove the air. Diaphragmatic hernia is an important diagnosis that presents in children and must be ruled out as a possible etiology.

## Abbreviations

ED: Emergency department
CT: Computed tomography.
